# Supplementary figures and images for: Pathogen and human NDPK-proteins promote AML cell survival via monocyte NLRP3-inflammasome activation
Source: PLoS One. 2023 Jul 7;18(7):e0288162. doi: 10.1371/journal.pone.0288162 (PMC10328239; doi:10.1371/journal.pone.0288162)

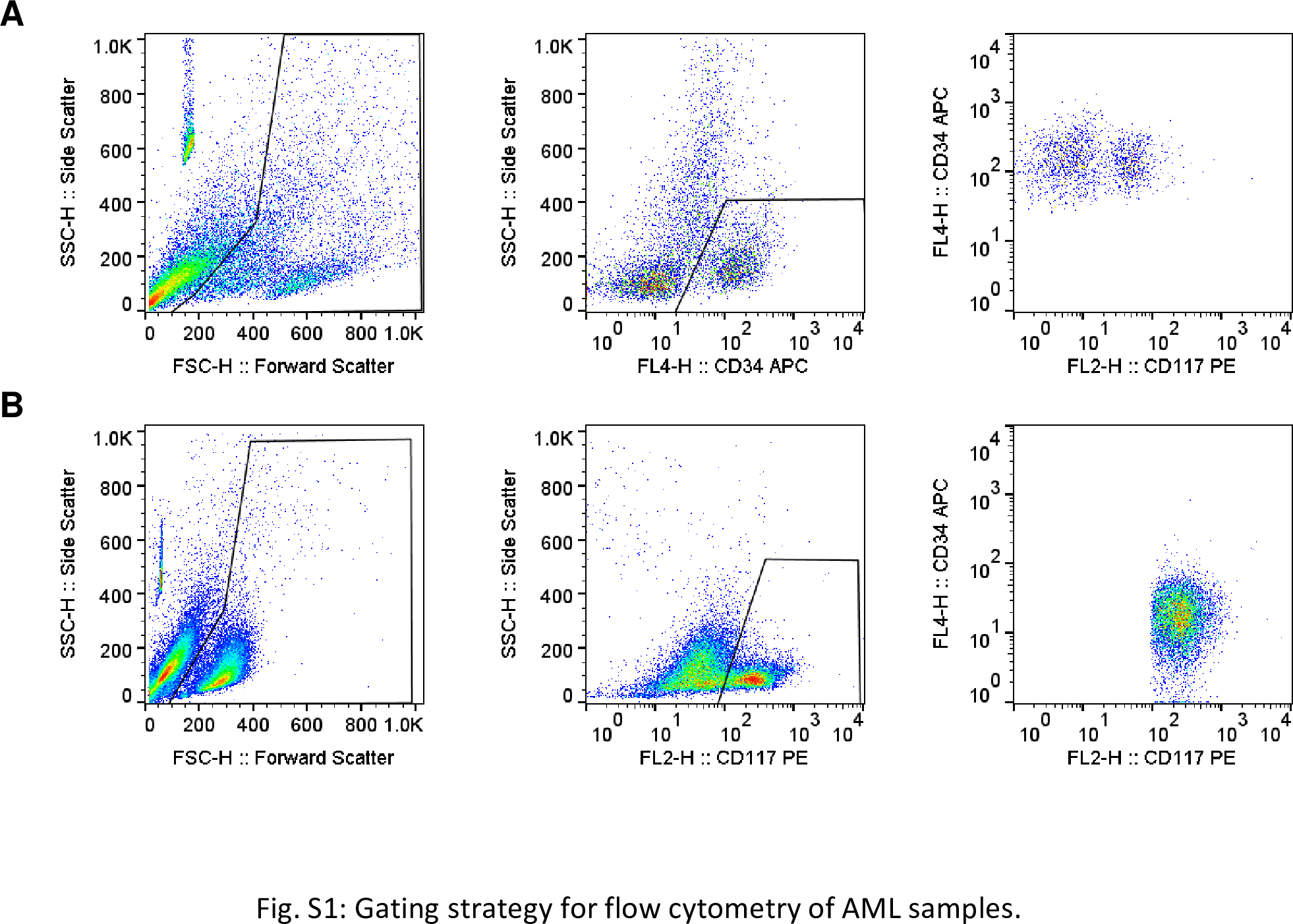

Supplement: S1 Fig — Two AML samples are shown as an example of the gating strategy. Live cells were identified by Forward Scatter/Side Scatter plot; blast cells were then identified by size and their main blast marker at diagnosis (either CD34 (A) or CD117 (B)). Finally, dual positivity for the second blast marker (either CD117 (A) or CD34 (B)) was verified with a CD117/CD34 plot. (TIF) [file pone.0288162.s001.tif]

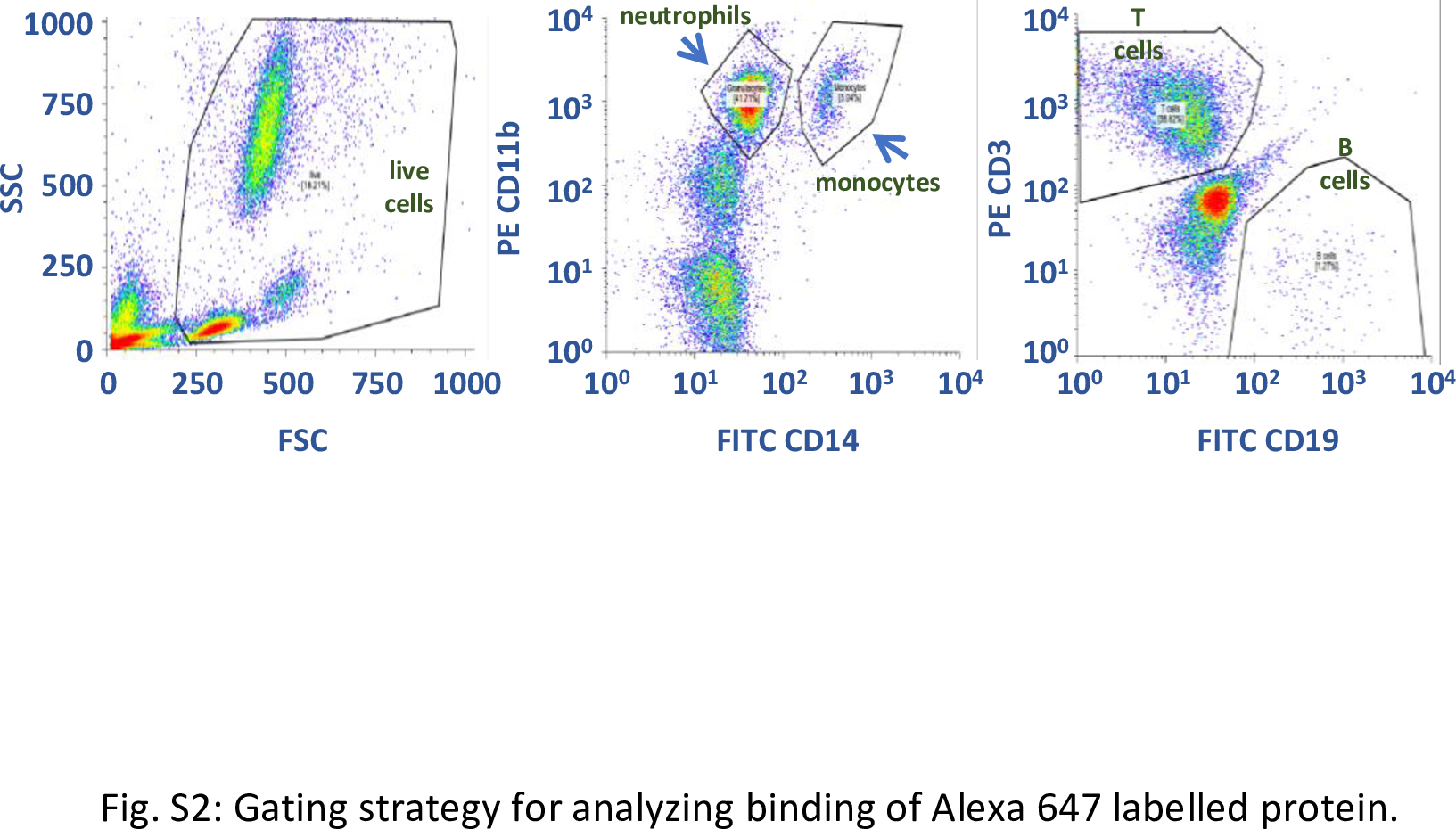

Supplement: S2 Fig — Left: Representative forward and side scatter plot of red-cell lysis prepared donor blood showing live gate. Centre: Neutrophils were identified as CD11b+CD14- and monocytes as CD11b+CD14+. Right: in a separate tube, T cells were identified as CD3+CD19- and B-cells as CD3+CD19-. (TIF) [file pone.0288162.s002.tif]

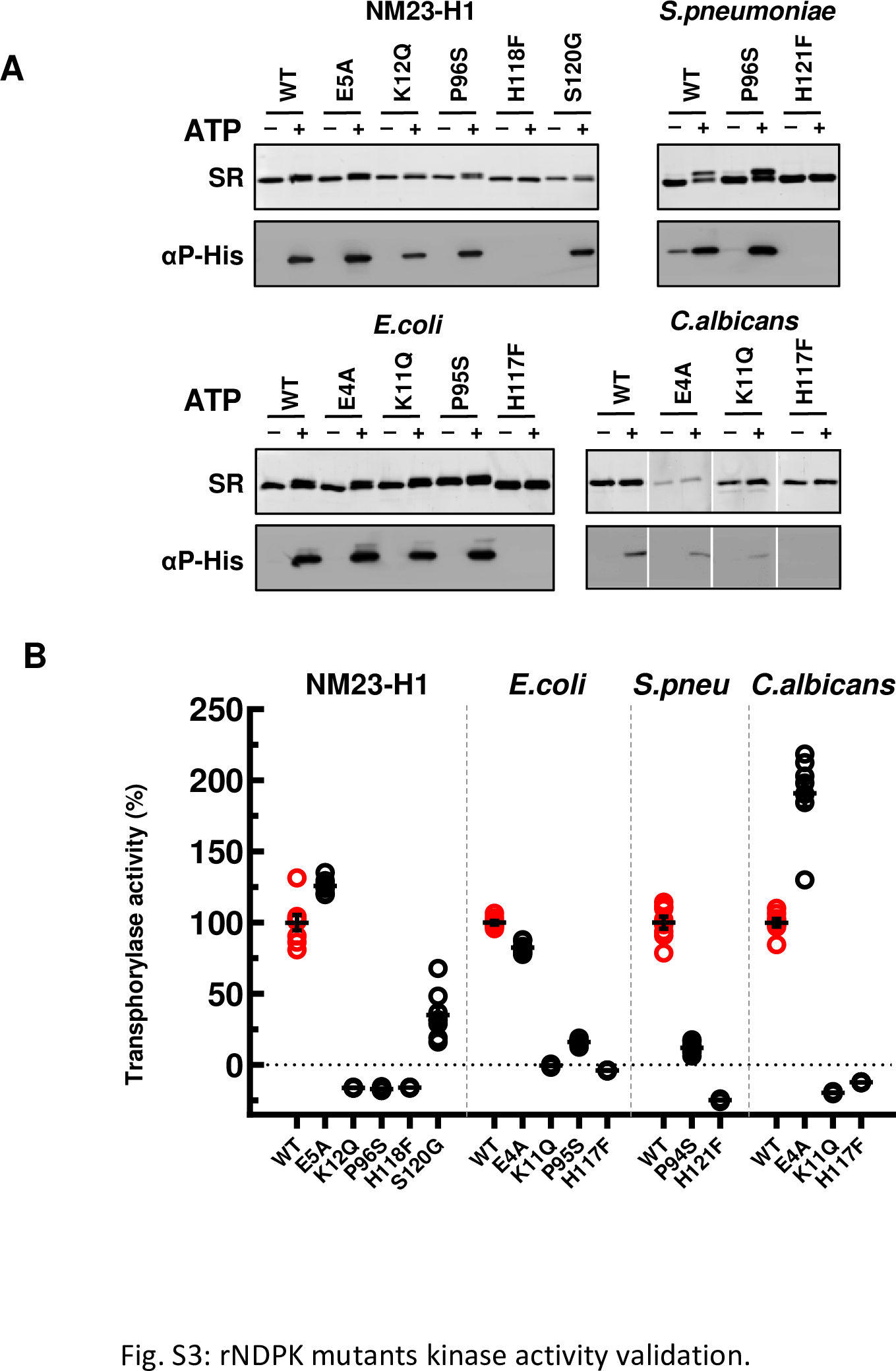

Supplement: S3 Fig — To create mutants all mutagenesis reactions were performed directly on the NDPKs sequences cloned in the pET15b backbone. Alignment amongst NM23-H1 and bacterial and fungal NDPK was performed with Clustal 2.1. This identified the conserved residues and allowed to infer, based on NM23-H1 literature, which of the residues would have impaired the enzyme function and structural organization on pathogen NDPK. Primers were designed with PrimerX online software (https://www.bioinformatics.org/primerx/) with design protocol for QuikChange Site-Directed Mutagenesis and codon optimized for E.coli expression systems. Mutagenesis reaction was performed using QuikChange II Site-Directed Mutagenesis kit (Agilent Technologies) following the manufacturer’s instructions. Mutations were confirmed by sequencing and the plasmid were transformed in BL21(DE3) for protein production. (A) Recombinant NDPKs, wild type (WT) and mutants, were pre-incubated in the presence or absence of ATP and run on two SDS-PAGE. One was stained with Sypro Ruby (SR) protein stain to detect total protein levels (on top) and the other was transferred and immunoblotted for an anti-N1-phosphohistidine antibody (αP-His). The autophosphorylation of rNDPKs was analyzed with ATP as substrate in the presence of MgCl2. 50ng of stock rNDPKs were incubated with and without 1mM ATP for 5 minutes at room temperature in 30μl buffer (20mM Tris-HCl, pH 8.0; 150mM NaCl and 1mM MgCl2). Reaction was stopped by adding 5mM EDTA for Mg2+ chelation. 10μl of 4X loading buffer (50% Glycerol; 10% β-Mercaptoethanol; 7.5% SDS; 300 mM Tris/HCl; pH 6.8; 0.25% Bromophenol blue) were added and proteins separated without heat denaturation on two 15% SDS-PAGE. The first gel was stained with SYPRO Ruby Protein Gel Stain (ThermoFisher Scientific) according to the manufacturer’s instructions. Proteins from the second gel were transferred onto a 0.2μm nitrocellulose membrane (Amersham Protran) and probed overnight at 4°C with 1:1000 αN1-Phosphohi [file pone.0288162.s003.tif]

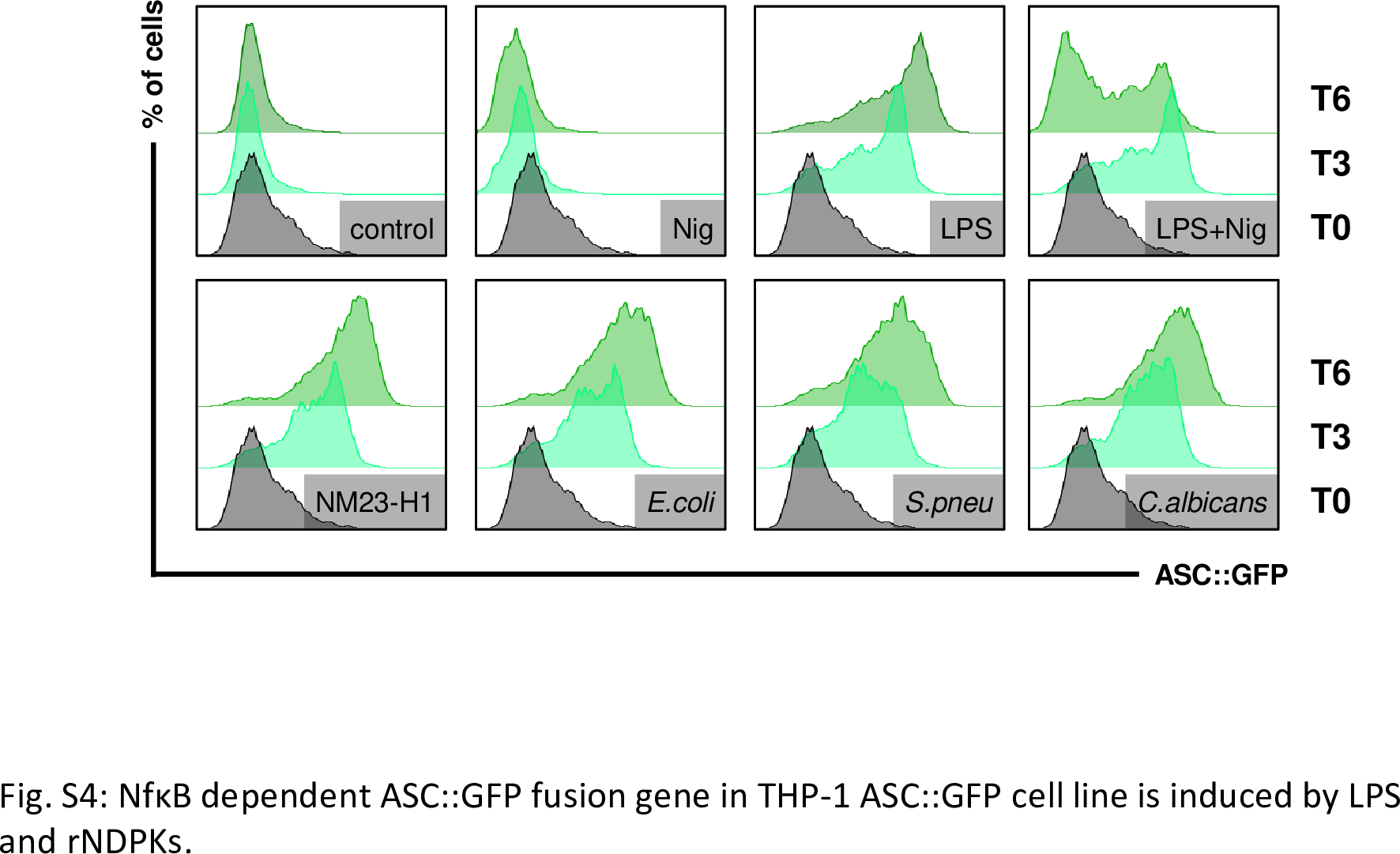

Supplement: S4 Fig — THP-1 ASC::GFP were treated for 3 and 6 hours with Nigericin 20μM, LPS and rNDPK 2μg/ml. In LPS+Nig treatment, was first induced for 3h with LPS 2μg/ml and then Nigericin 20μM was added to induce speck formation. T0 = untreated cells at time 0; T3 = 3 hours; T6 = 6 hours. (TIF) [file pone.0288162.s004.tif]

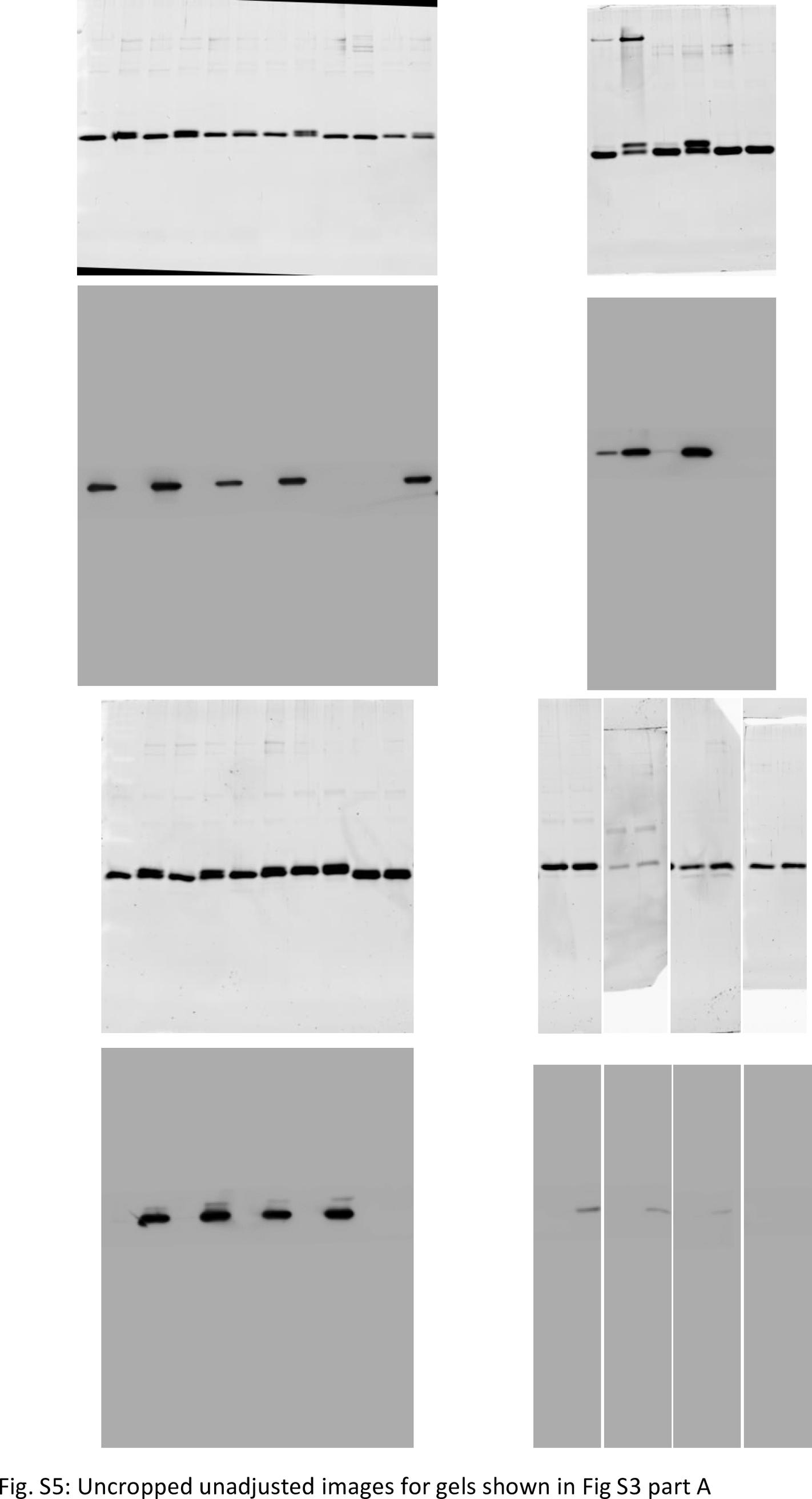

Supplement: S5 Fig — (TIF) [file pone.0288162.s005.tif]

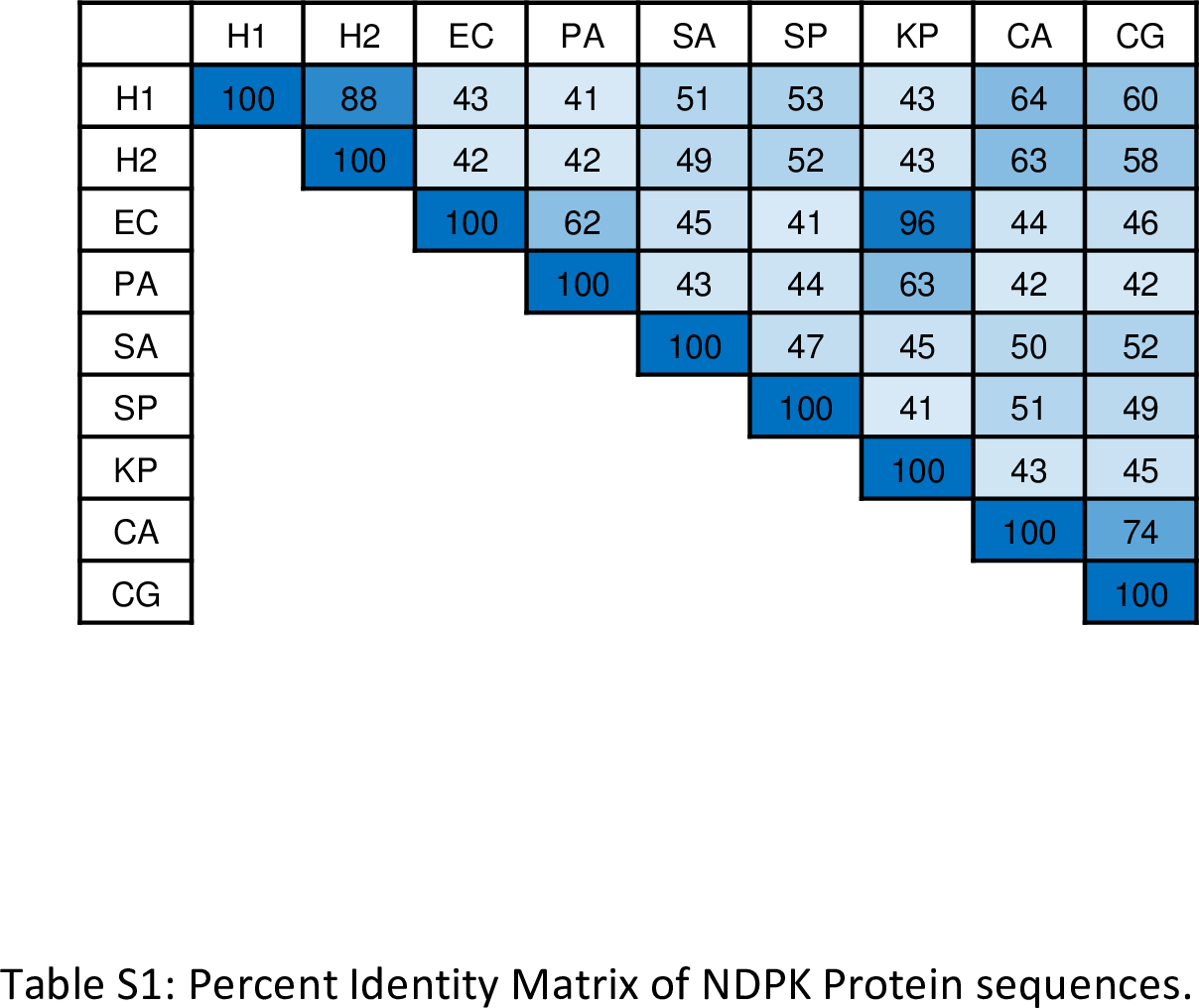

Supplement: S1 Table — NM23-H1 (H1), NM23-H2 (H2), Escherichia coli (EC), Pseudomonas aeruginosa (PA), Staphylococcus aureus (SA), Staphylococcus pneumoniae (SP), Klebsiella pneumoniae (KP), Candida albicans (CA), Candida glabrata (CG) were aligned and Percent Identity Matrix was generated with Clustal 2.1. (TIF) [file pone.0288162.s006.tif]

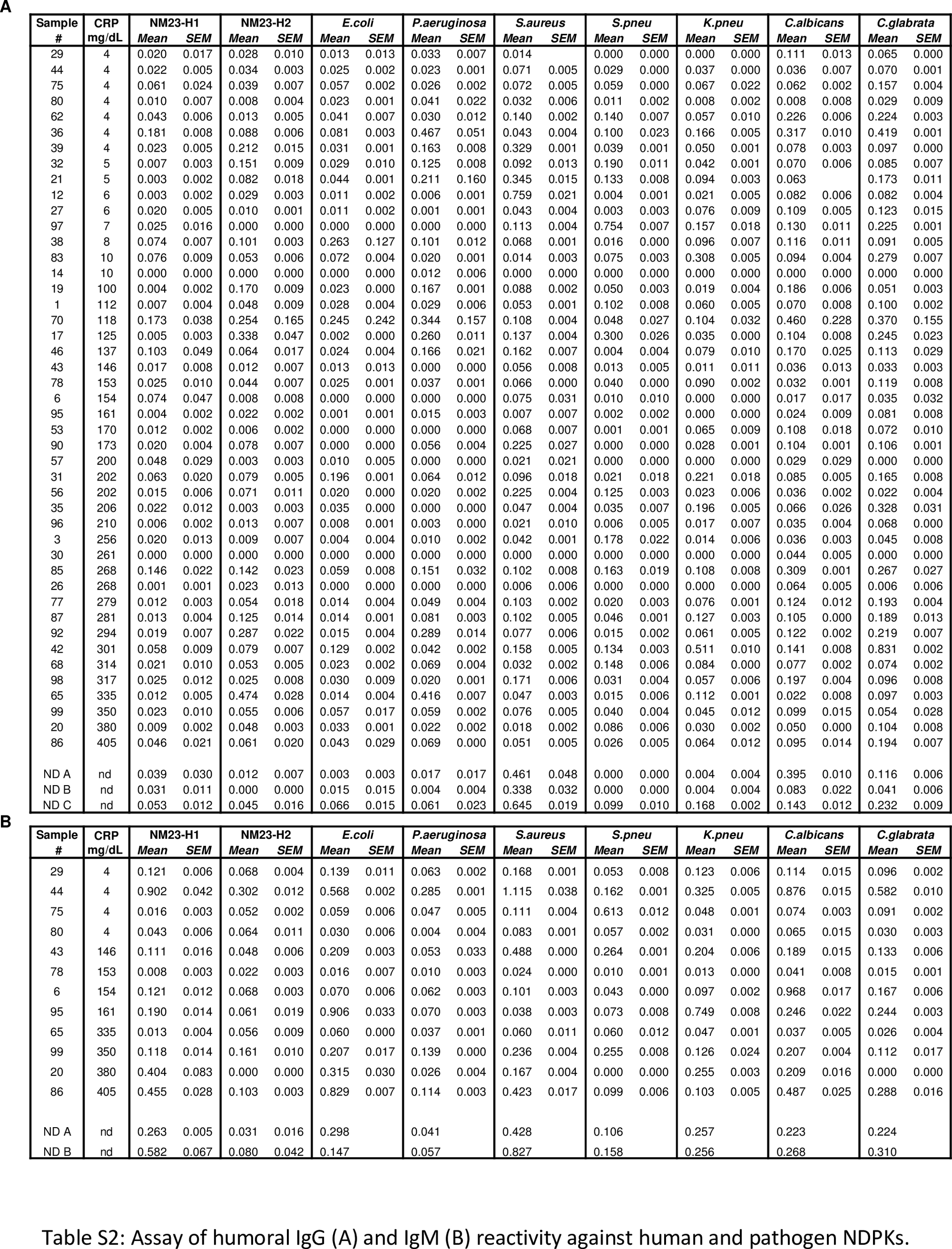

Supplement: S2 Table — Assay of humoral IgG (A) and IgM (B) reactivity against human and pathogen NDPKs. Table shows Individual data used to plot graphs in Fig 1C & 1D. AMLs were selected and ranked with a range of CRP (C-reactive protein) levels (mg/L). ND = normal donor; nt = not tested. As shown in the table all samples tested for IgM levels were also tested for IgG levels. (TIF) [file pone.0288162.s007.tif]

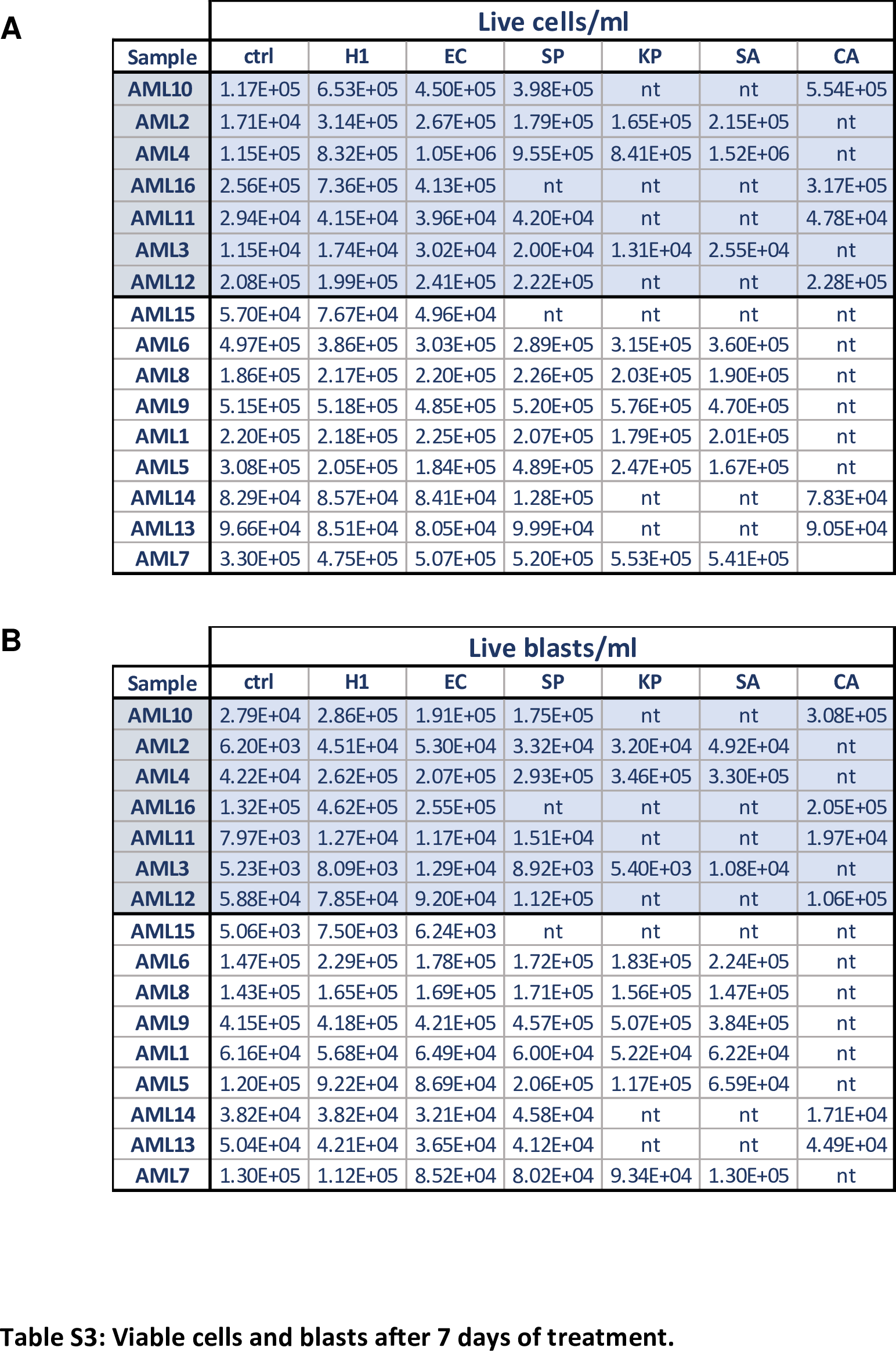

Supplement: S3 Table — Primary AML cells were plated out at 1x10^6^ cells/ml and treated with rNDPKs for 7 days. Viable total cells (A) and blasts (B) were enumerated by Flow cytometry and the use of counting beads and are presented in the Table. H1: rNM23-H1, EC: rNDPK E.coli; SP: rNDPK S.pneumoniae; KP: rNDPK K.pneumoniae; SA: rNDPK S.aureus; CA: rNDPK C.albicans. nt = not tested. (TIF) [file pone.0288162.s008.tif]

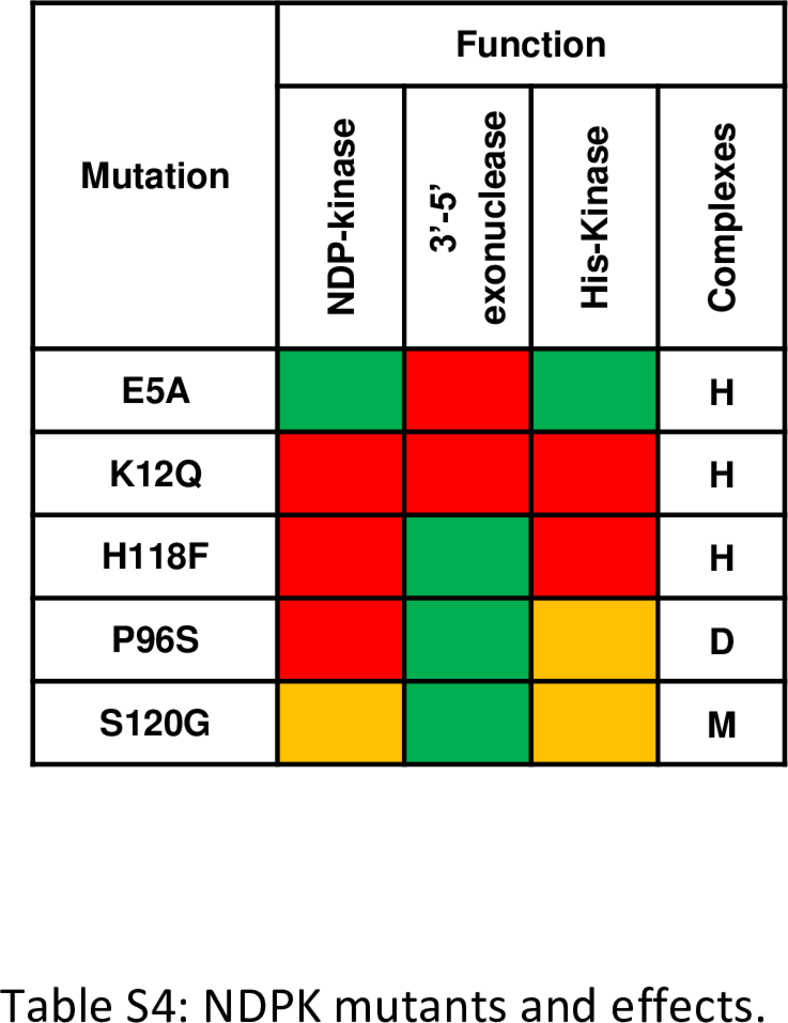

Supplement: S4 Table — NM23-H1 mutants and their effects. Green indicates retained function, yellow a moderate loss while red impaired activity. H: Hexamer; D: Dimer; M: Mix of dimeric and hexameric structures. E5A Glu-5 to Ala; K12Q Lys-12 to Gln; H118F His-118 to Phe, P96S Pro-96 to Ser, S120G Ser-120 to Gly. (TIF) [file pone.0288162.s009.tif]
