# Supplementary material for: Pathogen and human NDPK-proteins promote AML cell survival via monocyte NLRP3-inflammasome activation
Source: PLoS One. 2023 Jul 7;18(7):e0288162. doi: 10.1371/journal.pone.0288162 (PMC10328239; doi:10.1371/journal.pone.0288162)
Supplement: Attachment — Submitted filename: Response to Reviewers.docx [file pone.0288162.s010.docx]

Dear Dr Bertolini	02 June 2023

*PONE-D-23-03267: Pathogen and human NDPK-proteins promote AML cell survival via monocyte NLRP3-inflammasome activation.*

Thank you for reviewing our manuscript. I apologise that we did not quite manage to send our revision by May 28^th^. The lead Post-Doctoral researcher Dr Sandro Trova has since left the lab and as a result it has taken longer than we expected to revise the manuscript (in particular retracing original gel images to show uncropped).

As requested, we provide here a rebuttal that responds to each point raised by the academic editor and reviewer(s), a marked up copy of the manuscript highlighting changes made to the original version and an unmarked version of the new manuscript.

We do not wish to make changes to our financial disclosure and have removed any funding-related text from the manuscript.

We have endeavoured to ensure that the manuscript meets the style requirements for PLOS ONE and provided additional details regarding participant consent.

We have included tables as part of the main manuscript and included the captions for our Supporting Information files at the end of the manuscript.

We have included original gel images in the supplementary information.

Responses to reviewers’ comments

Reviewer #1:

Acute myeloid leukaemia (AML) and myelodysplastic syndromes MDS) are blood cancers that are associated with frequent infections. Experimental evidences are reported here that infections may promote or drive cancer progression.
The authors show that nucleoside diphosphate kinases (NDPK) from both Gram-positive (S. aureus, S. pneumoniae) and Gram-negative (E. coli, K. pneumoniae) bacteria and fungi (C. albicans), activate inflammatory mechanisms and promote AML cell survival.
NDPKs from pathogens have been shown to recapitulate the pro-survival effect of human NM23-H1 protein on AML cells, where NM23-H1 shows significant sequence similarity with NDPKs from pathogens.
These findings widen the understanding of the physio-pathological relationships likely existing between infections and tumor growth and progression.
The only concern that can rise at this stage is to make the Results and the Discussion more focused and better defined.

It is not easy to address this type of generic critique which is to some extent at odds with the fact that all three reviewers confirmed that the is manuscript presented in an intelligible fashion and written in standard English. As acknowledged in the manuscript, we asked a non-involved colleague to read the manuscript ahead of submission. We do not feel that there is need for any further rewriting of the repaper beyond the changes made in response to reviewer’s comments below.

Reviewer #2:

The paper from Trova S. et al. identifies a new role for pathogen NDPK-proteins in promoting survival of AML blasts through induction of an inflammatory response. These data raise the important implication that pathogen infections in AML patients could play an active role in the leukemogenic process, rather than being simply a consequence. I think that the paper is technically sound and clearly written. The experiments were conducted appropriately and the authors discuss their data in agreement with their findings.

However, since the inflammatory response induced by NDPK-proteins seems to be mediated mainly by normal monocytes, how can the authors explain that around 60% (9/16) of AML tested samples are not responders and do not show any survival advantage?

That is an interesting point. We do not know what distinguishes AML samples that respond and do not. However, it is most likely not variable monocyte responses between patients, but more likely differential responses to downstream consequences of the inflammatory microenvironment. For example, an ex vivo functional screen of 94 cytokines, identified that IL-1β elicited expansion of myeloid progenitors in some but not all AMLs (reference 7 in the manuscript)

Is there any correlation between response and previous pathogen infections of the AML patients from which blasts were derived, and/or the level of differentiation of the AML blasts, and/or any other clinical feature of AML?

We have addressed this question ourselves, and as far as we have such information, we have not found such correlations. We do not have infection histories for the patients whose samples we have analysed so we have not been able to address that question.

In response to this and the previous point by this reviewer we have added extra text to the discussion;

*“As seen in our previous studies and those of others the survival response of AML samples to NDPKs was selective. We have not observed eithewr positive or negative responses to be associated with any available clinical variable such as blast count or differentiation status of AML blasts. One explanation for the differential responses displayed by AML cells are differential responses to downstream consequences of inflammasome activation. For example, an ex vivo functional screen of 94 cytokines, identified that IL-1β elicited expansion of myeloid progenitors in some but not all AMLs (7)”*.

I also have a minor technical issue. In supplementary Figure S2, the blots showing total protein levels of the different mutants by Sypro Ruby staining are not visible. It is difficult to judge equal protein expression and loading.

We have enlarged the figure which makes the Sypro Ruby staining easier to see.

Reviewer #3:

Trova and colleagues describe in this manuscript the show that NDPKs from different strains of bacteria and one fungus are able to activate inflammatory mechanisms and promote AML cell survival in patients. They showed that the activation is mediated by monocyte and in cascade by neutrophils. Moreover, they showed that the release of IL-1beta depends on the activity of caspase-1.The manuscript is well written and all the experiments are logical and well described.
However this reviewer has some issues on the experiments:
Major revision:
1. In figure 1C and 1D the authors measured the IgM and IgG levels in different AMLs and healthy donors. They used for the IgM 14 AMLs and 2 Healthy donors, while for the IgG 45 AMLs and 3 Healthy donors. It is not clear why there is this discrepancy in the numbers between the two experiments, and why the authors did not use the same sample for IgG and IgM. The study would have been more interesting if within the same sample both IgG and IgM were measured
For the 12 AMLs and 2 healthy donors that we performed IgM ELISAs we also did IgG measurements. Having observed that the IgM response was more generalised and the IgG-response more selective, we then investigated IgG levels in a greater sample set. The comparative levels of IgM and IgG anti-NDPK antibodies are documented in supplementary table S2. We have altered the legend of both Fig1 and supplementary Table S2 to make this clearer.

To the Fig1 legend we have added “*All samples measured for IgM activity were also measured for IgG activity. CRP values and individual antibody activities for all samples are summarized in Table S2”.*

The legend to Table S2 now reads: “*Table shows Individual data used to plot graphs in Figure 1 C&D. AMLs were selected and ranked with a range of CRP (C-reactive protein) levels (mg/L). ND = normal donor; nt = not tested. As shown in the table all samples tested for IgM levels were also tested for IgG levels”*.

2. In Figure 2A FACs plot of cells are presented. Authors discriminates alive cells (red) and dead cells (black) by their morphology. This analysis seems to be too much arbitrary. It would have been better to use a marker of cell vitality (7-AAD, DAPI, etc.) and repeat the analysis. Moreover, it is not clear how the authors have counted the alive cells for the graph 2B.

The approach to live cell gating used in figure 2A, albeit not as sophisticated as ‘live cell’ stains is routine practice in laboratories around the world including those using flow cytometry in a diagnostic service setting. The common use of FSC and SCC to exclude debris and cell fragments is described on several web sites for companies supplying flow cytometry reagents

eg <https://www.bio-rad-antibodies.com/flow-cytometry-gating-strategies.html>

and

<https://www.stemcell.com/considerations-for-facs-gating.html>. Furthermore, fluorescent stains such as 7-AAD and DAPI undergo spectral shifts upon association with DNA they do not discriminate between live and dying cells (both contain DNA) and they also label apoptotic fragments that contain DNA.

The live counting of cells in Figure 2B has been made possible by including of counting beads in the assay tubes (see page 15 lines 382-383 and Figure 2b legend lines line 646).

3. It would be useful in experiments showed in Figure 3A, to show how cells are subdivided in B cells, T cells, monocytes, and neutrophils. A suggestion might be showing FACS plot subdividing firstly the different subpopulations and then with the positivity towards fluorescent NDPK
This is a very helpful suggestion we have now provided a summary of the gating strategy employed as Supplementary Fig. S2

4. It would be useful to show FACS plot of monocytes depleted samples in Figure 3D, to demonstrate the purity of isolated monocytes

We concede that it would have been useful to some readers to demonstrate the depletion of monocytes in samples. However, we did not perform these analyses and cannot show the data. That said, monocyte depletion was carried out using widely used standard commercially available and robust purification kits that deploy CD14 monoclonal antibody conjugated magnetic bead technologies. The responses are severely diminished post monocyte depletion.

5. In Figure 5 authors claimed to have used THP1 deficient or knockout for caspase1. It would be useful to show a western blot indicating the lack of the protein.

The important variable for measuring inflammasome activity is levels of caspase 1 activity not levels of protein *per se*. We used caspase deficient THP-1 cells from InvivoGen. These cells are not completely null for caspase 1, but have an approximately 7-fold reduction in protein levels as described by the supplier. Consistent with this we show in Figure 5B that basal unstimulated caspase 1 activity in these cells was substantially lower than in control THP1 cells. When stimulated with Nigericin control THP-1 cells generated more than 10 times the caspase 1 activity generated in Caspase 1 deficient cells. In response to LPS control cells generated 5-7 fold more Caspase 1 activity than Caspase 1 deficient cells. Similar differentials in Caspase-1 activity between control and caspase-1 deficient cells were seen in response to human, bacterial and yeast NDPKs. Because the cells were caspase deficient and not null, we further utilised the caspase-1 inhibitor to test the dependency of nigericin, LPS and human, bacterial and fungal NDPKs on inflammasome activation as measured by secretion of IL-1β.

Minor comments:
1. Table 1 should be put in order after Figure 1

This is now the case

2. Immunofluorescence images are completely out of focus. They should be acquired better

An EVOS instrument was used to acquire those images of live cells not confocal microscopy. Thus, the figures appear less clear. However, the images are more than adequate to show the result. What the reader needs to take from panel A is that few cells are expressing ASC::GFP protein in the control. On exposure to LPS, NM23-H1 or NDPKs from *E.coli* and *S.pneu,* many more cells express ASC::GFP in a diffuse cytoplasmic distribution. In contrast nigericin together with LPS resulted in the LPS-induced ASC::GFP expression becoming aggregated in discrete structures indicative of pyroptosome generation. Similarly in panel B, ASC immuno-staining shows greater frequency of pyroptosome ‘specks’ in response to nigericin alone than the other treatments.
